# The role of complementary lymphadenectomy in patients with incidental endometrial cancer

**DOI:** 10.3389/fonc.2025.1635672

**Published:** 2025-10-22

**Authors:** Salim Abraham Barquet-Muñoz, Pamela Martínez-Alpizar, Andrea Ramirez, Pamela Rico-Mejía, Delia Pérez-Montiel, Cintia María Sepúlveda-Rivera, Jonathan González-Ruiz, Alejandro Mohar, Carlos Pérez-Plasencia, David Cantú-de-León, Diddier Prada

**Affiliations:** ^1^ Departamento de Ginecología, Instituto Nacional de Cancerología, Mexico City, Mexico; ^2^ Unidad de Apoyo Molecular para la Investigación Clínica, Instituto Nacional de Cancerología, Mexico City, Mexico; ^3^ Departamento de Patología, Instituto Nacional de Cancerología, Mexico City, Mexico; ^4^ Departamento de Ginecología Oncológica, Instituto Nacional de Perinatología, Mexico City, Mexico; ^5^ Unidad de Investigación Biomédica en Cáncer, Instituto Nacional de Cancerología. Instituto de Investigaciones Biomédicas, UNAM, Mexico City, Mexico; ^6^ Laboratorio de Genómica, Instituto Nacional de Cancerología, Tlalpan, Laboratorio de Genómica Funcional, Unidad de Biomedicina, Facultad de Estudios Superiores (FES) Iztacala (FES-IZTACALA), Universidad Nacional Autónoma de México, Tlalnepantla, Mexico City, Mexico; ^7^ Subdirección de Investigación Básica, Instituto Nacional de Cancerología, Mexico City, Mexico; ^8^ Institute for Health Equity Research, Department of Health Science and Policy and the Department of Environmental Medicine and Public Health, Icahn School of Medicine at Mount Sinai, New York City, NY, United States

**Keywords:** endometrial cancer, lymphadenectomy, complementary, prognosis, hysterectomy

## Abstract

**Background:**

Up to 3.0% of women are diagnosed with endometrial cancer after hysterectomy for apparently benign conditions. There is controversy about the benefit of complementary lymphadenectomy in incidental endometrial cancer after hysterectomy.

**Objective:**

To evaluate the role complementary lymphadenectomy during a second surgery in the prognosis of patients with endometrial carcinoma.

**Study design:**

This was a retrospective cohort study of patients who were diagnosed with endometrial carcinoma from 2005 to 2019. Two groups were evaluated: patients who underwent a second surgery involving pelvic and/or para-aortic lymphadenectomy and patients who did not undergo surgical lymph node evaluation. Logistic regression was used to identify the factors associated with whether or not a complementary lymphadenectomy was performed. The Kaplan–Meier method was used to generate survival curves, and the log-rank test was used for comparisons. Univariate and multivariate analyses were performed with the Cox test.

**Results:**

Two hundred and sixty patients were included. Among them, 120 (46.15%) underwent complementary lymphadenectomy, and 140 (53.83%) did not. The factors associated with performing complementary lymphadenectomy in a second surgical procedure were higher grade, nonendometrioid histology and deep myometrial involvement. The factors associated with adjuvant treatment were high-grade histology, deep myometrial involvement, cervical involvement and extensive lymphovascular permeation. Complementary lymphadenectomy was not associated with adjuvant treatment (OR 0.85 95% CI 0.35-2.02), overall survival (Hazard Ratio (HR) 0.40 95% CI 1.16-1.00) or disease-free survival (HR 0.77 95% CI 0.38-1.59).

**Conclusions:**

No clear therapeutic or prognostic role was identified for complementary lymphadenectomy during a second surgery in patients with endometrial cancer. Although adjuvant therapy was more common in patients who underwent complementary lymphadenectomy, it was not independently associated with receiving adjuvant therapy. Individualizing treatment decisions remains important when considering a second surgical procedure.

## Highlights

- Incidental endometrial cancer is diagnosed in up to 3% of women undergoing hysterectomy for benign conditions. The benefit of complementary lymphadenectomy during a second surgery remains controversial due to limited and inconsistent evidence.
- This study shows that complementary lymphadenectomy in a second surgical procedure is not associated with improved overall or disease-free survival in patients with incidental endometrial carcinoma. However, it is more likely to be performed in patients with aggressive pathological features and is associated with a higher likelihood of receiving adjuvant therapy.
- The findings support a more individualized approach in deciding on second-stage lymphadenectomy after incidental endometrial cancer diagnosis. Routine complementary lymphadenectomy may be unnecessary in the absence of high-risk features, which can influence clinical decision-making and avoid overtreatment.

## Introduction

Endometrial carcinoma is the second most common gynaecological malignancy in terms of incidence and the third most common cause of mortality worldwide, and in Mexico, the incidence of endometrial carcinoma in the last decade has increased (1, 2). It is estimated that by 2050, the incidence and mortality of endometrial carcinoma will increase by 676.3 thousand and 183.1 thousand, respectively (1). In 75% of patients, endometrial carcinoma is diagnosed in the early stages with an excellent prognosis, but in 20-25% of patients, there may be pelvic lymph node involvement, which is associated with decreased overall survival (OS) and disease-free survival (DFS) (3, 4).

The standard for assessing nodal involvement is pathologic analysis, with the procedure being identification of the sentinel node or pelvic and/or para-aortic lymphadenectomy. In this context, the role of lymphadenectomy is prognostic because it provides information for assessing the need for adjuvant treatment and can modify surgical stage by 10% (5, 6). Lymphadenectomy does not improve DFS (HR, 1.25, 95% CI 0.93-1.66; p=0.14) or OS (HR, 1.04, 95% CI 0.74-1.45; p=0.83); therefore, lymphadenectomy alone has no therapeutic role (7).

After receiving a hysterectomy for apparently benign conditions, 3.0% of woman may be diagnosed with endometrial carcinoma without undergoing lymph node evaluation (8). The benefit of complementary lymphadenectomy in a second surgical procedure is controversial (6, 9–11). The recommendation for patients with endometrial carcinoma, which is identified incidentally at hysterectomy for benign causes, is to complete surgical staging when there is suspicion or risk of extrauterine disease (12, 13).

The aim of this study was to evaluate the role of complementary lymphadenectomy in the prognosis of patients with endometrial carcinoma. The secondary objective was to establish the factors associated with the performance of complementary lymphadenectomy in an second surgical procedure lymphadenectomy.

## Methods

This was a retrospective cohort in which information was obtained from the electronic files of patients treated at the Instituto Nacional de Cancerología de México, México City, between January 1, 2005, and December 31, 2019. The records of women who underwent initial simple hysterectomy, either outside of or at the institution, and who had an incidental diagnosis of endometrial carcinoma, without initial surgical lymph node evaluation, were included.

The inclusion criteria were records of women with endometrial carcinoma who underwent initial simple hysterectomy outside the oncological institution for a presume benign pathology and needed follow-up or adjuvancy in the oncologic institution; and patients who underwent surgery at the oncologic institution but did not undergo lymphadenectomy due to a comorbidity or technical difficulty. The decision to perform the complementary lymphadenectomy was made by a multidisciplinary oncology board based on the findings of the pathology review, the patient’s morbidity (e.g., morbid obesity), and the fact that less than 3 months had passed since the surgery was performed outside the institution.

Patients who had not undergone hysterectomy, who had received neoadjuvant treatment, or who had second primary tumours were excluded. The clinical, pathological, surgical and adjuvant treatment variables of the patients were evaluated. Patients were divided into two groups. The first group, referred to as the complementary lymphadenectomy group, included individuals who underwent at a second surgical procedure, no more than 3 months after the first surgery, for pelvic and/or para-aortic lymphadenectomy within the limits established in the literature. The second group, referred to as the group without lymphadenectomy, included patients who did not undergo surgical lymph node evaluation.

### Statistical analysis

OS was defined as the period between diagnosis and death or the last visit, and disease-free survival was defined as the period between hysterectomy and recurrence or the last visit. A central tendency analysis was performed with medians and interquartile ranges (IQRs) for continuous variables and absolute and relative frequencies for qualitative variables. To perform the comparative analysis between the two groups, the Wilcoxon rank-sum test, the chi-square test or Fisher’s exact test was used according to the variable type. To identify the factors associated with the performance of complementary lymphadenectomy, logistic regression was used to calculate odds ratios (ORs). Survival curves were generated with the Kaplan–Meier method and compared with the log-rank test. Factors associated with survival were analysed with the Cox test. A significant difference was defined as a p value < 0.05. Statistical analysis was performed with the statistical program STATA version 16.0 (TX, USA). The study was approved by the Research and Ethics Committee of the National Cancer Institute (Reference INCAN/CI/0408/2022).

## Results

A total of 901 files were reviewed. Among them, 31 were excluded because the patient did not undergo surgery as the initial treatment, and 610 were excluded because the patient underwent lymph node evaluation during the initial surgery. After these exclusions, 260 files were included for further analysis. Of the patients described in these files, 120 (46.15%) underwent complementary lymphadenectomy, and 140 (53.83%) did not undergo this procedure.

The median age was 55 years (IQR 45.7-62.4). In total, 62 patients (23.85%) with suspicious nodes were identified by imaging, and among them. In the pathological evaluation of patients who underwent complementary lymphadenectomy, 31 (25.83%) had nodal involvement; among these patients, 22 had pelvic involvement, and 9 had both pelvic and para-aortic involvement. No patients had only para-aortic disease. There were 166 (63.85%) patients with stage I disease, 39 (15.0%) with stage II disease and 55 (21.15%) with stage III disease. In the comparison between the complementary lymphadenectomy group and the group without lymphadenectomy, there were significant differences in histological type (p<0.001), myometrial involvement (p<0.001), adnexal involvement (p<0.001), extensive lymphovascular space invasion (p=0.010), clinical stage (p<0.001), radiotherapy (p=0.001) and chemotherapy (p<0.001) (**Table 1**).

**Table 1:** Comparative analysis between complementary and non-complementary lymphadenectomy group.

Variable	Total	Complementary	Non-complementary	p
	260 (100%)	120 (46.15)	140 (53.85)	
Age	55 (45.7-62.4)	54.49 (46.51-59.66)	55.15 (45.01-62.44)	0.532
Menopause	201 (77.31)	92 (76.67)	109 (77.86)	0.819
BMI	29.78(26.07-34.63)	28.76 (26.33-31.22)	28.89 (25.97-34.22)	0.274
Lymph nodes in Image				
Negative	173 (66.54)	76 (63.33)	97 (69.29)	0.441
Positive	62 (23.85)	33 (27.50)	29 (20.71)	
NA	25 (9.62)	11 (9.17)	14 (10.0)	
Histology				
Endo G1	59 (22.69)	14 (11.67)	45 (32.14)	<0.001
Endo G2	134 (51.54)	68 (56.67)	66 (47.14)	
Endo G3	38 (14.62)	18 (15.0)	20 (14.29)	
PPH	29 (11.15)	20 (16.67)	9 (6.43)	
Myometrial involvement				
< 50%	103 (39.62)	31 (25.83)	72 (51.43)	<0.001
≥ 50%	122 (46.92)	68 (56.67)	54 (38.57)	
Unknown	35 (13.46)	21 (17.50)	14 (10.0)	
Cervical involvement				
Negative	202 (77.69)	89 (74.17)	113 (80.71)	0.073
Positive	55 (21.15)	31 (25.83)	24 (17.14)	
Unknown	3 (1.15)	0 (0)	3 (2.14)	
Serosal involvement				
Negative	238 (91.54)	109 (90.83)	129 (92.14)	0.644
Positive	12 (4.62)	5 (4.17)	7 (7.0)	
Unknown	10 (3.85)	6 (5.0)	4 (2.86)	
Adnexal involvement				
Negative	223 (85.77)	109 (90.83)	114 (81.43)	<0.001
Positive	18 (6.92)	11 (9.17)	7 (5.0)	
Unknown	19 (7.31)	0 (0)	19 (13.57)	
Pelvic nodes involved	31 (11.92)	31 (25.83)	0 (0)*	<0.001
Para-aortic nodes involved	9 (3.46)	9 (7.50)	0 (0)*	<0.001
Parametrial involvement				
Negative	250 (96.15)	117 (97.50)	133 (95.0)	0.56
Positive	4 (1.54)	1 (0.83)	3 (2.14)	
Unknown	6 (2.31)	2 (1.67)	4 (2.86)	
LVSI	81 (31.15)	47 (39.17)	34 (24.29)	0.010
Stage				
I	166 (63.85)	62 (51.67)	104 (74.29)	<0.001
II	39 (15.0)	19 (15.83)	20 (14.29)	
III	55 (21.15)	39 (32.50)	16 (11.43)	
Radiotherapy	179 (68.85)	95 (79.17)	84 (60)	0.001
Chemotherapy	78 (30.00)	53 (44.17)	25 (17.86)	<0.001

BMI, Body Mass Index; NA, Not performed; Endo, Endometroid; PPH, Poor Prognosis Histology; LVSI, Lymphovascular Space Invasion.

The independent factors associated with performing complementary lymphadenectomy (**Table 2**) were grade 2 endometrioid histology (OR 2.95, 95% CI 1.33-6.56; p=0.008), poor prognosis histology (OR 9.37, 95% CI 2.94-29. 86; p<0.001), and myometrial involvement greater than 50% or unknown (OR 2.75, 95% CI 1.49-5.07; p=0.001). Conversely, adnexal involvement was a factor associated with a lower likelihood of undergoing a second surgical procedure (OR 0.27, 95% CI 0.11-0.66; p=0.004). In the group of patients who underwent complementary lymphadenectomy, 108 (90%) were approached by laparotomy and 12 (10%) by laparoscopy, 10 (8.33%) patients had a recognized intraoperative injury, of which 1 (0.83%) required the suspension of surgery. The median bleeding was 200 ml (IQR 100–400), with a median hospital stay of 3 days (IQR 3–4). Six (5.56%) patients had immediate postoperative complications requiring some type of intervention in the immediate postoperative period.

**Table 2:** Factors associated with complementary lymphadenectomy.

	Univariate analysis			Multivariate analysis		
	OR	CI 95%	p	OR	CI 95%	p
Age	0.99	0.97-1.02	0.503	0.97	0.94-1.00	0.071
Menopause	0.93	0.52-1.67	0.819	0.81	0.36-1.80	0.612
BMI	0.961	0.92-1.01	0.109	0.97	0.92-1.02	0.326
Lymph nodes in Image	1.31	0.78-2.19	0.311	1.22	0.79-1.88	0.365
Histology						
Endo G1	Reference					
Endo G2	3.31	1.66-6.59	0.001	2.95	1.33-6.56	0.008
Endo G3	2.89	1.21-6.94	0.017	1.98	0.69-5.70	0.203
PPH	7.14	2.66-19.21	<0.001	9.37	2.94-29.83	<0.001
Myometrium ≥ 50%/Unknown	3.04	1.79-5.15	<0.001	2.75	1.49-5.07	0.001
Cervical Involvement/Unknown	1.46	0.81-2.62	0.207	0.90	0.45-1.78	0.766
Serosal involvement/Unknown	1.18	0.49-2.84	0.706	0.76	0.26-2.14	0.606
Adnexal Involvement/Unknown	0.44	0.21-0.94	0.034	0.27	0.11-0.66	0.004
Parametrial Involvement/Unknown	0.49	0.12-1.93	0.305	0.33	0.68-1.66	0.182
LVSI	2.01	1.18-3.42	0.010	1.55	0.79-3.05	0.199

BMI, Body Mass Index; NA, Not performed; Endo; Endometroid; PPH, Poor Prognosis Histology; LVSI, Lymphovascular Space Invasion.

The independent factors associated with receiving some type of adjuvant treatment are presented in **Table 3**. Age (OR 1.05, 95% CI 1.00-1.09; P = 0.037), grade 3 endometrioid histology (OR 15.65, 95% CI 2.67-91.67; P = 0.002), poor prognosis histology (OR 7.06, 95% CI 3.01-16.57; P<0.001), myometrial involvement ≥50% or unknown (OR 7. 06 95% CI 30.1-16.57; p<0.001), cervical or unknown involvement (OR 19.45, 95% CI 3.94-95.91; p<0.001) and lymphovascular space invasion (OR 4.19 95% CI 1.42-12.38; p=0.009) were significant. Lymphadenectomy was associated with a higher risk for adjuvant treatment in the univariate analysis (OR 2.97 95% CI 1.67-5.27; p<0.001) but not in the multivariate analysis (OR 0.85, 95% CI 0.35-202; p=0.716).

**Table 3:** Factors associated to adjuvant treatment (RT or Chem or Both).

	Univariate			Multivariate		
	OR	IC 95%	p	OR	IC 95%	p
Age	1.03	1.01-1.06	0.011	1.05	1.00-1.09	0.037
Menopause	1.39	0.75-2.58	0.287	0.46	0.14-1.50	0.203
BMI	0.96	0.91-1.01	0.083	1.00	0.94-1.06	0.959
Lymph nodes in Image/Unknown	1.09	0.62-1.93	0.752	0.68	0.29-1.59	0.384
Histology						
Endo G1	Reference					
Endo G2	5.74	2.95-11.15	<0.001	2.29	0.95-5.51	0.064
Endo G3	35.1	7.66-160.89	<0.001	15.65	2.67-91.67	0.002
PPH	16.9	4.56-62.69	<0.001	10.66	2.00-56.86	0.006
Myometrial ≥ 50%/Unknown	8.02	4.39-14.65	<0.001	7.06	3.01-16.57	<0.001
Cervical Involvement/Unknown	16.89	4.01-71.2	<0.001	19.45	3.94-95.91	<0.001
Serosal Involvement/Unknown	4.69	1.06-20.59	0.041	0.96	.014-6.61	0.974
Adnexal Involvement/Unknown	1.02	0.47-2.17	0.969	1.13	0.35-3.68	0.831
Parametrial Involvement/Unknown	1.75	0.36-8.43	0.487	0.87	0.91-8.31	0.906
LVSI	6.95	3.03-15.95	<0.001	4.19	1.42-12.38	0.009
Complementary lymphadenectomy	2.97	1.67-5.27	<0.001	0.85	0.35-2.02	0.716
Lymph Node Involvement	15.19	2.03-113.55	0.008	5.79	0.62-54.15	0.123

RT, Radiotherapy; Chem, Chemotherapy; Both, Chemotherapy plus Radiotherapy BMI, Body Mass Index; NA, Not performed; Endo, Endometroid; PPH, Poor Prognosis Histology; LVSI, Lymphovascular Space Invasion; OR, odds ratio; CI 95%, Confidence Interval 95%.

**Figure 1:**
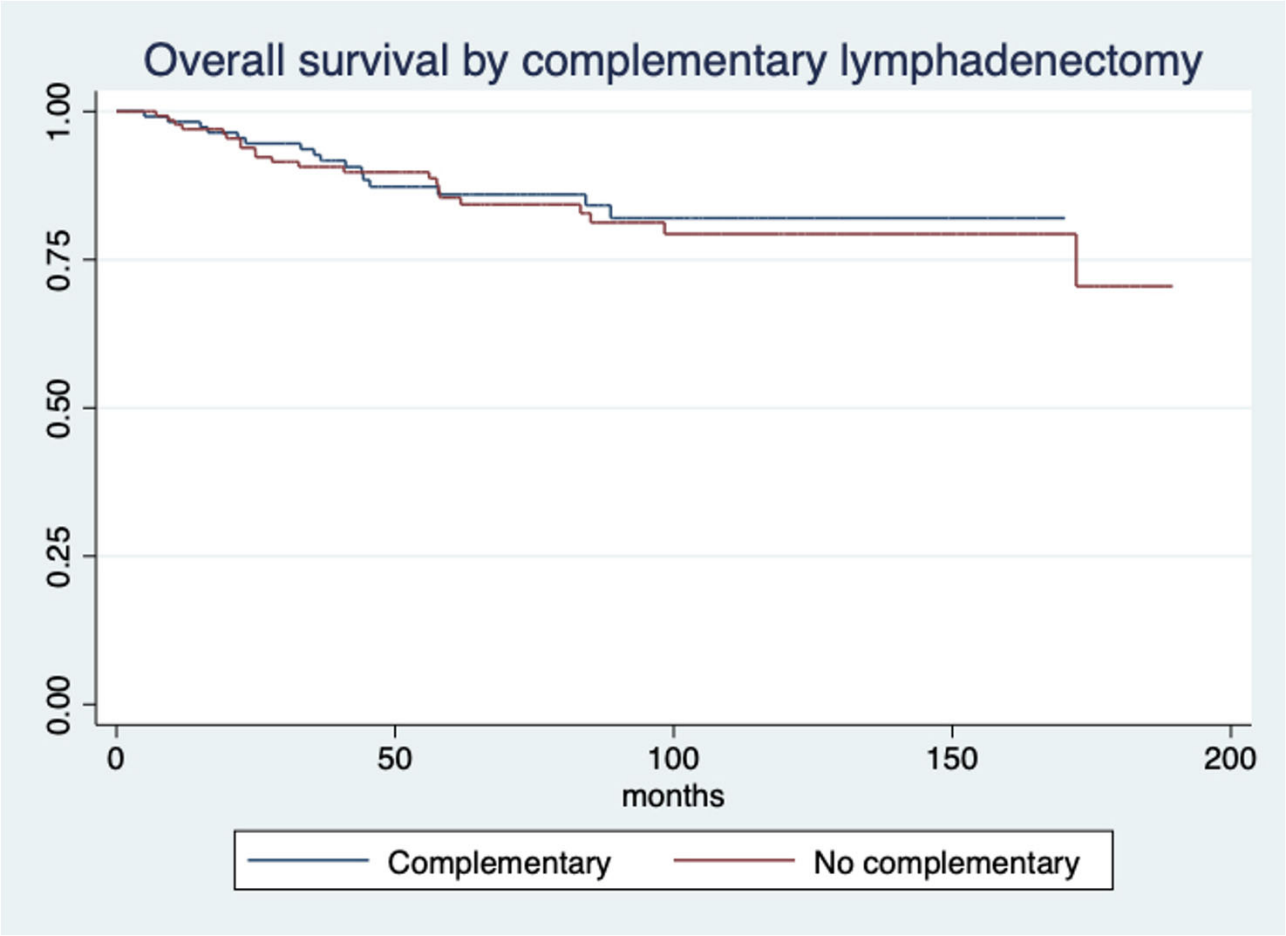
Kaplan-Meier curve for overall survival at 5 years by group of treatment in patients with treated at the National Cancer Institute of Mexico. The significance test between groups was obtained by the log-rank test.

**Figure 2:**
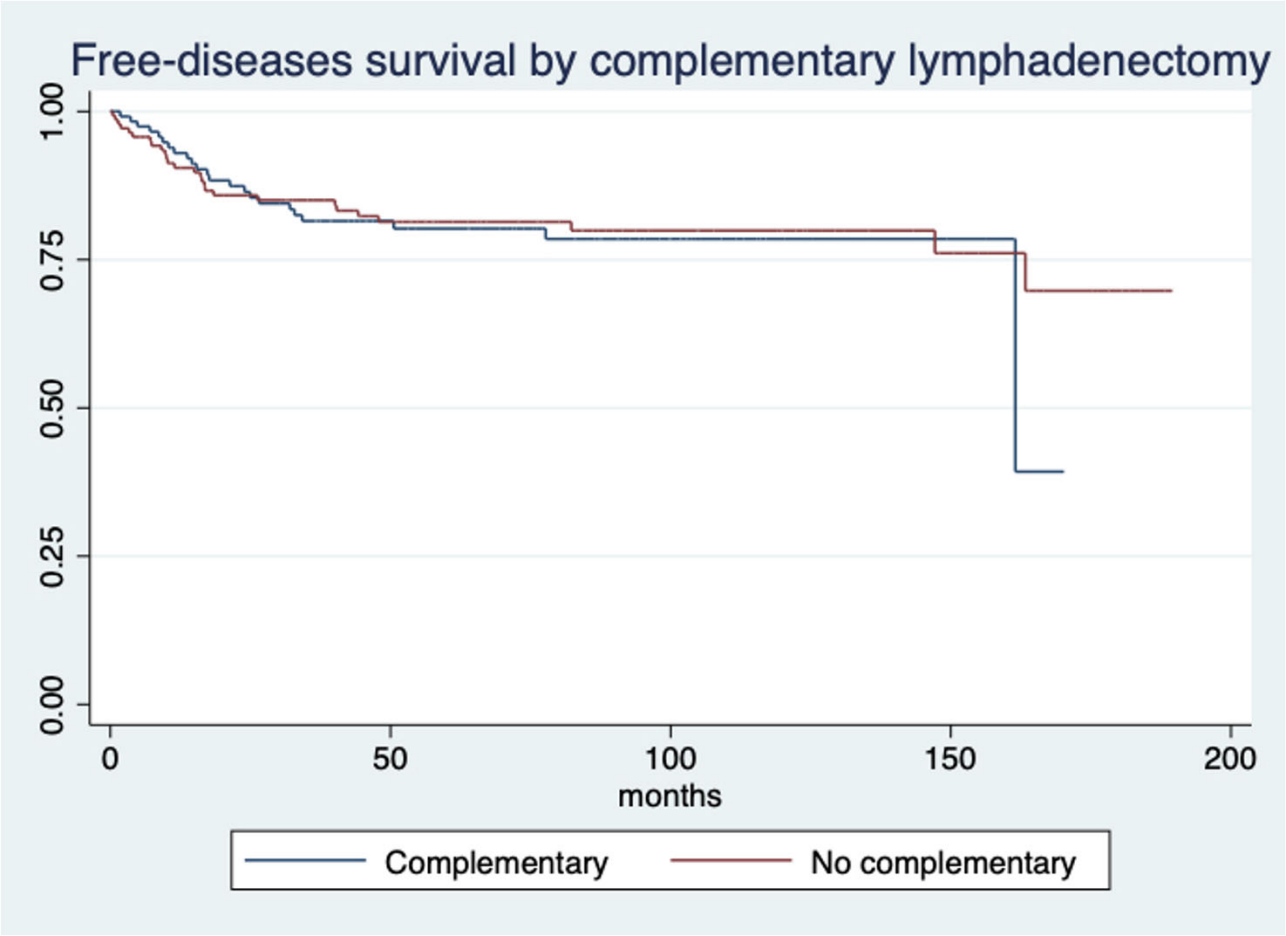
Kaplan-Meier curve for free-disease survival at 5 years by group of treatment in patients with treated at the National Cancer Institute of Mexico. The significance test between groups was obtained by the log-rank test.

The median follow-up time was 67.2 months (IQR 37.4-102.83). The five-year OS rate was 85.69% (95% CI 80.17-89.76%), and the five-year disease-free survival rate was 80.83% (95% CI 75.13-85.36). The 5-year OS rate of the complementary lymphadenectomy group was 86.01% (95% CI 77.39-91.52), and that of the group that did not undergo a second surgery was 85.47% (95% CI 77.50-90.78); this difference was not significant (p=0.753) (**Figure 1**). The five-year DFS rate in the complementary lymphadenectomy group was 80.25% (95% CI 71.24-86.70), and that in the group that did not undergo a second surgery was 81.73% (95% CI 73.44-78.15), which was not significantly different (p=0.744) (**Figure 2**).

Moreover, complementary lymphadenectomy was not independently associated with OS (HR 0.40, 95% CI 0.16-1.00; p=0.051) or DFS (HR 0.77 95% CI 0.38-1.54; p=0.462) (**Supplementary Tables S1**, **S2**, respectively).

## Discussion

### Summary of main results

The results indicate that in patients with endometrial cancer, complementary lymphadenectomy performed as a second surgical procedure does not correlate with improved OS or DFS. This outcome stems from the fact that lymphadenectomy serves as a prognostic rather than a therapeutic entity and is used primarily to pathologically assess lymph node involvement and determine the need for adjuvant treatment. In the group undergoing complementary lymphadenectomy, 25% of the patients had lymph node involvement. The primary purpose of lymphadenectomy is to establish lymph node involvement to guide subsequent adjuvant management. However, nodal involvement was not the sole factor influencing the decision for adjuvant treatment in our cohort. Other clinical and pathological factors from the initial hysterectomy, such as age, poor prognosis histology, low differentiation grade, cervical involvement, greater depth of invasion, and lymphovascular space invasion, were also considered.

### Results in the context of published literature

In a study by Ayhan et al. involving 40 patients, complementary lymphadenectomy resulted in 20% upstaging without significant differences in OS (88.89% vs. 84.62%, p>0.05) or DFS (95.24% vs. 87.50%, p>0.05) (9). Similarly, Panici et al. reported that among 514 patients with endometrial carcinoma, lymphadenectomy altered the tumour stage by 10.1% (13.3% vs. 3.2%, 95% CI 5.3%-14.9%, p<0.001) but did not improve OS (81.0% vs. 85.9%) or DFS (81.7% vs. 90.0%) (6). Another study from 1970 to 2006 with 581 patients reported a 25% increase in staging upon final pathological evaluation, with no improvement in OS (HR 1.00, p=0.992) or DFS (HR 0.96, p=0.815) compared to patients who did not undergo lymphadenectomy (10). Goudge et al. highlighted that among 291 completely staged patients, 18% had their disease stage changed, and 21 received adjuvant radiotherapy or chemotherapy (11).

International guidelines sometimes recommend complementary lymphadenectomy. The National Comprehensive Cancer Network (NCCN) advises it for patients with endometrioid histology staged below III, those with suspicious lymph nodes by imaging, or those at high risk of nodal involvement. It is not deemed essential for low-risk patients without suspicious lymph node involvement. For nonendometrioid histologies, there is no recommendation. European Society for Medical Oncology (ESMO) suggests complementary lymphadenectomy for incompletely staged patients or those at intermediate or high risk, especially if it may alter adjuvant treatment plans (12–15).

### Implications for practice and future research

Factors associated with performing complementary lymphadenectomy include poor differentiation, poor prognosis, and deep or unknown myometrial involvement, especially when lymph node involvement is unknown by imaging. Adnexal involvement is a factor against complementary lymphadenectomy, as it independently indicates the need for adjuvant chemotherapy and radiotherapy regardless of nodal status.

Notably, complementary lymphadenectomy in a second surgical procedure did not independently influence the decision for adjuvant treatment, as these patients often had other poor prognostic factors. Nevertheless, a higher proportion of these patients received adjuvant radiotherapy or chemotherapy. It is important to note that surgical procedures are not free of complications that can delay adjuvant management; although the percentage of immediate intraoperative and postoperative complications was low in this series, it should be considered that these can delay treatment with radiotherapy or chemotherapy, if they occur. The prognostic value of nodal status in endometrial cancer is well established; however, when retroperitoneal staging is performed as a complementary lymphadenectomy after an incidental diagnosis, its therapeutic role remains controversial. In this setting, the potential prognostic benefit of detecting nodal disease must be carefully weighed against the surgical risks and morbidity associated with a second procedure. This highlights the need to individualize management decisions and to consider less invasive alternatives, such as sentinel node mapping, particularly in patients with comorbidities or limited surgical tolerance (6, 16, 17).

Evidence supports the benefit of adjuvant therapy based on postoperative findings, independent of nodal evaluation. A study of 3,664 patients with apparent early-stage endometrial cancer who did not undergo lymphadenectomy but received adjuvant radiotherapy showed a better 5-year DFS (89.9% vs. 87.8%, p=0.04), particularly in patients under 70 years of age, with grade 3 disease, and with stage II disease. Factors independently associated with DFS included age, clinical stage, and histologic grade (18, 19). Additionally, lymphadenectomy can influence adjuvant decisions, as seen in a study of 349 patients, where 12% received adjuvant treatment and 17% were able to avoid radiotherapy or chemotherapy based on surgical staging results (20). Currently, the role of lymph node evaluation in apparently early-stage endometrial cancer is solely prognostic and not therapeutic. Sentinel lymph node examination is sometimes used as an alternative to lymphadenectomy due to its lower risk of complications and even a higher positivity rate when using ultra staging (21, 22).

### Strengths and weaknesses

A strength of our study is its focus on the role of complementary lymphadenectomy in a second surgical procedure; an area not extensively covered in recent literature. The study is limited by its retrospective, non-randomized design. Confounding by indication may have influenced the results, particularly when analysing patients who did not undergo complementary lymphadenectomy and had worse prognosis. Also, we acknowledge that molecular features such as *p53* status or mismatch repair (MMR) deficiency, which are increasingly relevant in endometrial cancer, were not available in this retrospective cohort. This omission limits the ability to align our findings with the most recent molecular classifications. In addition, advanced statistical approaches such as propensity score analysis were not applied, which could have further controlled for residual confounding. Finally, there may be variability in adjuvant treatment protocols, as well as occasional unavailability of complete hysterectomy specimens for thorough review.

## Conclusions

No clear therapeutic or prognostic role was identified for complementary lymphadenectomy during a second surgery in patients with endometrial cancer. Although adjuvant therapy was more common in these patients, it was not independently associated with receiving adjuvant therapy, which may reflect that complementary lymphadenectomy is often performed in patients with poor prognostic factors. However, independently, poor prognostic factors, such as nonendometrioid histology, poor differentiation grade, and cervical involvement, are the factors that influence the decision to provide adjuvant treatment in this group of individuals who lack a complete surgical staging procedure or who undergo lymph node evaluation at the time of initial surgery. Despite these results, it is important to address each patient individually to make the best decision possible regarding surgical reintervention.

## Data Availability

The raw data supporting the conclusions of this article will be made available by the authors, without undue reservation.
