# Supplementary material for: The role of complementary lymphadenectomy in patients with incidental endometrial cancer
Source: Front Oncol. 2025 Oct 22;15:1635672. doi: 10.3389/fonc.2025.1635672 (PMC12585951; doi:10.3389/fonc.2025.1635672)
Supplement: Supplementary file 1 [file Table1.docx]

| Supplementary table 1. Factors associated to Overall Survival | | | | | | | |
| --- | --- | --- | --- | --- | --- | --- | --- |
| Variable | Univariate | | | Multivariate | | |  |
|  | HR | IC 95% | p | HR | IC 95% | p |  |
| **Age** | 1.04 | 1.01-1.07 | 0.013 | 1.06 | 1.02-1.10 | 0.002 |  |
| **Menopause** | 1.36 | 0.59-3.08 | 0.468 | 0.72 | 0.25-2.03 | 0.540 |  |
| **BMI** | 0.98 | 0.92-1.04 | 0.504 | 1.01 | 0.96-1.08 | 0.540 |  |
| **Lymph nodes in Image / Unknown** | 2.78 | 1.47-5.27 | 0.002 | 2.79 | 1.35-5.73 | 0.005 |  |
| **Histology** |  |  |  |  |  |  |  |
| **Endo G1** | Reference |  |  |  |  |  |  |
| **Endo G2** | 3.01 | 0.87-10.41 | 0.081 | 1.82 | 0.45-7.31 | 0.395 |  |
| **Endo G3** | 8.14 | 2.23-29.77 | 0.002 | 4.47 | 0.91-21.87 | 0.064 |  |
| **PPH** | 7.16 | 1.84-27.86 | 0.004 | 3.65 | 0.73-18.33 | 0.115 |  |
| **Myometrial ≥ 50%/ Unknown** | 1.82 | 0.90-3.67 | 0.095 | 1.36 | 0.58-3.16 | 0.47 |  |
| **Cervical Involvement/ Unknown** | 2.24 | 1.16-4.33 | 0.017 | 2.75 | 1.26-6.00 | 0.011 |  |
| **Serosal Involvement/ Unknown** | 2.13 | 0.83-5.48 | 0.116 | 1.35 | 0.47-3.87 | 0.567 |  |
| **Adnexal Involvement / Unknown** | 2.06 | 0.94-4.51 | 0.071 | 2.56 | 1.01-6.47 | 0.046 |  |
| **Parametrial Involvement / Unknown** | 2.42 | 0.58-10.10 | 0.224 | 1.67 | 0.29-9.60 | 0.562 |  |
| **LVSI** | 2.63 | 1.38-5.02 | 0.003 | 1.11 | 0.48-2.56 | 0.804 |  |
| **Complementary lymphadenectomy** | 0.90 | 0.47-1.73 | 0.753 | 0.40 | 0.16-1.00 | 0.051 |  |
| **Lymph Node Involvement** | 2.62 | 1.23-5.55 | 0.012 | 1.99 | 0.67-5.92 | 0.214 |  |
| **Radiotherapy** | 2.32 | 0.97-5.55 | 0.058 | 0.66 | 0.20-2.17 | 0.495 |  |
| **Chemotherapy** | 3.98 | 2.07-7.65 | <0.001 | 2.11 | 0.80-5.55 | 0.128 |  |
| **BMI:** Body Mass Index, NA: Not performed, Endo: Endometroid, PPH: Poor Prognosis Histology, LVSI: Lymphovascular Space Invasion, HR: Hazard Ratio, CI 95%: Confidence Interval 95%. | | | | | | |  |
