# Supplementary material for: The role of complementary lymphadenectomy in patients with incidental endometrial cancer
Source: Front Oncol. 2025 Oct 22;15:1635672. doi: 10.3389/fonc.2025.1635672 (PMC12585951; doi:10.3389/fonc.2025.1635672)
Supplement: Supplementary file 2 [file Table2.docx]

| Supplementary table 2. Factors associated to Disease Free Survival | | | | | | |
| --- | --- | --- | --- | --- | --- | --- |
| Variable | Univariate | | | Multivariate | | |
|  | HR | IC 95% | p | HR | IC 95% | p |
| **Age** | 1.02 | 0.99-1.04 | 0.253 | 1.03 | 0.99-1.06 | 0.066 |
| **Menopause** | 0.91 | 0.48-1.72 | 0.774 | 0.60 | 0.26-1.36 | 0.226 |
| **BMI** | 0.98 | 0.93-1.03 | 0.410 | 0.99 | 0.94-1.05 | 0.973 |
| **Lymph nodes in Image / Unknown** | 1.92 | 1.09-3.36 | 0.022 | 1.78 | 0.97-3.28 | 0.062 |
| **Histology** |  |  |  |  |  |  |
| **Endo G1** | Reference |  |  |  |  |  |
| **Endo G2** | 2.17 | 0.93-5.10 | 0.074 | 1.31 | 0.49-3.48 | 0.583 |
| **Endo G3** | 2.74 | 0.99-7.52 | 0.051 | 1.46 | 0.42-5.09 | 0.547 |
| **PPH** | 2.81 | 0.98-8.05 | 0.054 | 1.30 | 0.35-4.79 | 0.690 |
| **Myometrial ≥ 50%/ Unknown** | 1.93 | 1.04-3.58 | 0.037 | 1.60 | 0.76-3.36 | 0.209 |
| **Cervical Involvement/ Unknown** | 1.98 | 1.10-3.55 | 0.023 | 1.75 | 0.89-3.44 | 0.104 |
| **Serosal Involvement/ Unknown** | 2.59 | 1.16-5.79 | 0.020 | 1.43 | 0.58-3.49 | 0.431 |
| **Adnexal Involvement / Unknown** | 1.90 | 0.95-3.83 | 0.070 | 1.83 | 0.83-4.07 | 0.133 |
| **Parametrial Involvement / Unknown** | 2.42 | 0.75-7.81 | 0.139 | 1.88 | 0.44-7.98 | 0.389 |
| **LVSI** | 2.24 | 1.28-3.93 | 0.005 | 1.42 | 0.69-2.91 | 0.337 |
| **Complementary lymphadenectomy** | 1.09 | 0.63-1.93 | 0.744 | 0.77 | 0.38-1.54 | 0.462 |
| **Lymph Node Involvement** | 1.97 | 0.95-4.07 | 0.066 | 1.43 | 0.56-3.67 | 0.450 |
| **Radiotherapy** | 1.77 | 0.89-3.55 | 0.016 | 0.86 | 0.35-2.12 | 0.746 |
| **Chemotherapy** | 1.96 | 1.11-3.44 | 0.020 | 1.24 | 0.56-2.72 | 0.582 |
| **BMI:** Body Mass Index, NA: Not performed, Endo: Endometroid, PPH: Poor Prognosis Histology, LVSI: Lymphovascular Space Invasion, HR: Hazard Ratio, CI 95%: Confidence Interval 95%. | | | | | | |
